# Shape factor analysis as a quantitative framework for assessing spheroid and organoid morphology and invasiveness

**DOI:** 10.1063/5.0333152

**Published:** 2026-07-01

**Authors:** Brittany E. Schutrum, Jenny Deng, Ju Hee Kim, Amalie Gao, Emily Hur, Jack C. Crowley, Lu Ling, Matalin G. Pirtz, Coulter Q. Ralston, Alexander Yu. Nikitin, Claudia Fischbach

**Affiliations:** 1 Nancy E. and Peter C. Meinig School of Biomedical Engineering, Cornell University, Ithaca, New York 14850, USA; 2 School of Applied Engineering Physics, Cornell University, Ithaca, New York 14853, USA; 3 Department of Biomedical and Translational Sciences, Cornell University, Ithaca, New York 14853, USA

## Abstract

Morphological changes in spheroids and organoids are widely used as *in vitro* indicators of healthy and diseased tissue function, but selecting appropriate methods to quantify these changes remains challenging. Shape factors (or shape descriptors) are dimensionless metrics often computed using ImageJ/FIJI; however, their ability to classify specific morphological features can vary. To address this challenge, we developed a clinically inspired, custom MATLAB algorithm to quantify the variance in radial lengths of invasive protrusions in spheroids and organoids. We then compared the advantages and limitations of this approach with conventional ImageJ/FIJI shape descriptors to guide users in selecting the most appropriate method for classifying spheroid and organoid morphology in their specific settings. To this end, we first analyzed digital phantoms and then performed the same comparisons using images from experimental spheroid and organoid datasets. By enabling numerical morphological readouts, shape factor analysis can enhance phenotypic profiling of spheroids and organoids and provide valuable metrics for *in vitro* studies, including high-throughput and drug screening workflows.

## INTRODUCTION

Advanced 3D model systems have emerged as critical tools to recapitulate the complex microenvironment of normal and malignant tissues in a controlled experimental environment.^1–3^ Breast cancer progression is routinely studied using spheroids or organoids embedded into extracellular matrix mimetics.^4–7^ Using these models, malignant progression can be estimated by analyzing time-dependent changes in spheroid/organoid shape, size, and margins using microscopy images. However, identifying tractable metrics to objectively quantify *in vitro* spheroid and organoid morphology is challenging. Given the emerging interest in spheroids^8^ and organoids^9^ for precision medicine and drug discovery, there is a widespread need for morphology image analysis that is adaptable for data collection in high-throughput settings. The development of such techniques may benefit from approaches currently used in the clinic.

Routine imaging-based screening has contributed to making breast cancer the most diagnosed cancer in women worldwide.^10^ Although primary tumors can be treated relatively effectively, invasive cancer is associated with a worse clinical prognosis due to its increased likeliness to metastasize to distant organs.^11^ Therefore, much emphasis has been placed on detecting cancer cell invasion early and developing therapies to interfere with this process.^11^ During screening, irregular tumor margins are a primary focus of radiologists' qualitative evaluation, as they are indicative of local invasion and thus, represent a key prognostic indicator of malignancy.^12–14^ To improve diagnostic accuracy, radiologist readings are complemented by computer-aided detection software^15,16^ that uses machine learning-based technology to highlight suspicious regions for radiologist review. Inspired by the value of computationally assistive tools in the clinic, we sought to develop a similar approach to quantify the morphology and invasion of 3D-cultured breast cancer spheroids in research settings and benchmark this metric against a subset of other frequently used analytic techniques.

Previous approaches to classify *in vitro* spheroid invasion range from simple analysis of spheroid size^4^ to advanced machine learning algorithms.^17,18^ The most straightforward methods measure the largest invasion distance from the core of a spheroid or quantify the change in area over time.^4,19^ While these metrics can classify the presence of invasion, they cannot differentiate a growing, noninvasive spheroid from an invasive spheroid. With high-resolution imaging, single-cell invasion can be quantified by segmenting the core of the spheroid and measuring the invasion distance of individual cells.^20^ Similarly, collective invasion analyses have focused on measuring the number and dimensions of collective invasion strands.^21–23^ However, these collective and single-cell analysis methods rely on acquiring time-consuming high-resolution images and often additional staining, processes that are impractical in high-throughput settings. Characterizing a basic shape factor, such as circularity, from lower-resolution images of spheroids/organoids has been implemented to circumvent these challenges,^24^ and tools such as OrganoSeg can segment brightfield images and quantify shape factors in a single workflow.^25,26^ Yet, these approaches also come with limitations as the frequently used shape descriptors in ImageJ/FIJI^27^ or CellProfiler^28^ conflate invasion with other morphological features, including elongation, asymmetric invasion, or regions of concavity. Advances in machine learning can address these limitations,^18^ but widespread adoption is still limited due to the need for specialized software and knowledge. Ultimately, there is a wide variety of tools and metrics to analyze spheroid/organoid morphology, but use of each is limited by the end user's application, scale, imaging data quality, and level of computational experience.

To fill this gap, we developed a custom MATLAB code for radial length analysis of spheroid and organoid morphology that was initially developed to identify perimeter undulations or surface irregularities of tumors in clinical images.^29^ It is designed to quantify variations in perimeter contours, represented as radial lengths, in a size-agnostic manner, thus allowing for comparisons across different length scales. For shape factor quantification and comparisons, we use custom-designed digital phantoms and experimental datasets. This approach identified key advantages and limitations of each metric and provides a guide for users to select the most appropriate metrics for their specific experiments. Using this approach, we found radial length analysis to be advantageous for radially symmetric collectively invading spheroids, while circularity or convexity was better suited to characterize organoids with folds or protrusions. Overall, this work highlights how multivariate shape factor analysis can be implemented for reliable and comprehensive quantification of spheroid and organoid morphologies using low-resolution bright-field images.

## RESULTS

### Advantages and limitations of standard FIJI shape factor analysis to quantify invasion

**FIG. 1.**
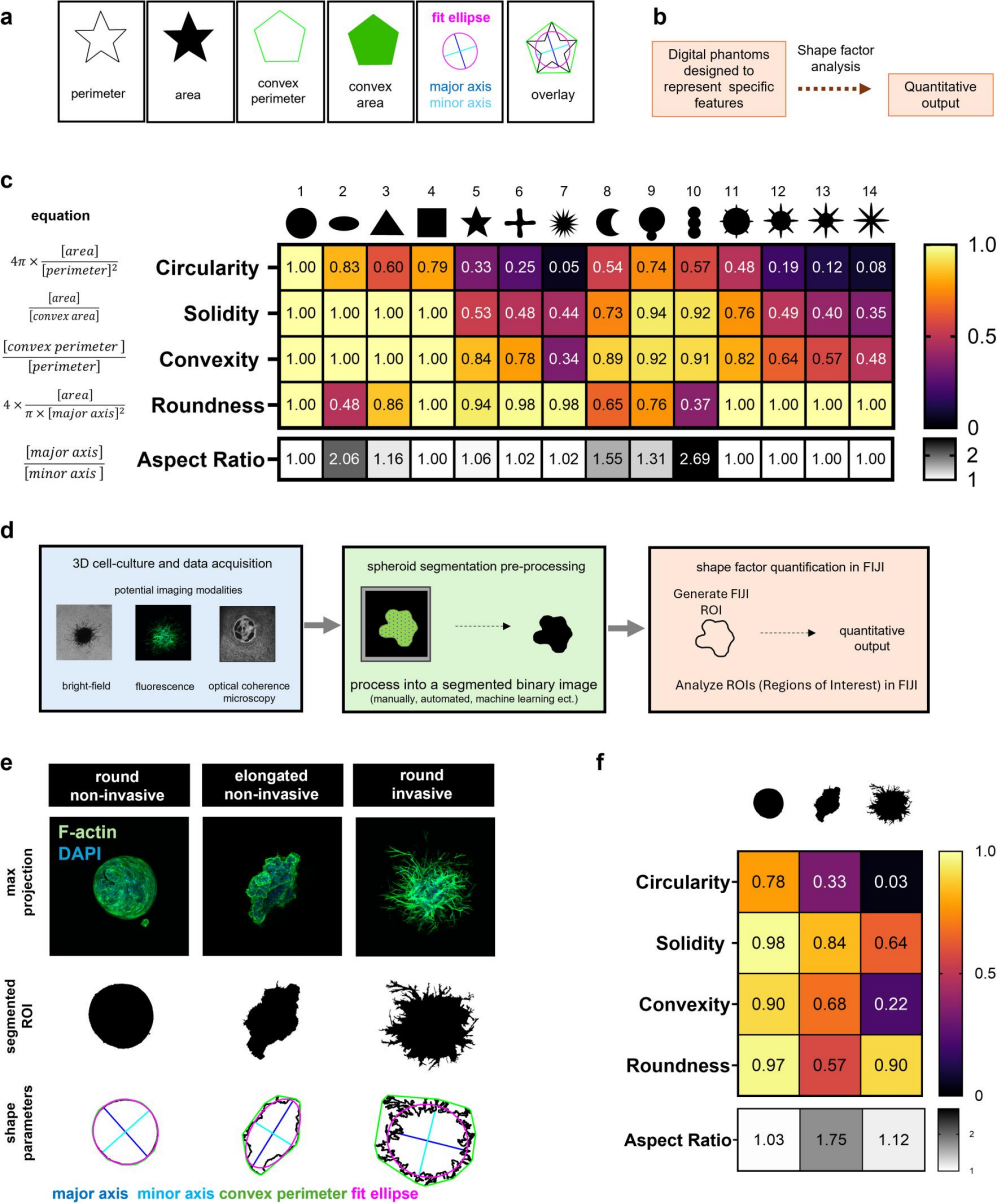
Analysis of FIJI/ImageJ shape descriptors to quantify morphological changes of digital phantoms and spheroids in experimental settings. (a) Schematic of a star shaped digital phantom to highlight component metrics used to calculate FIJI shape descriptors. (b) Schematic depicting digital phantom use. (c) Heat maps visualize the capabilities of each metric to differentiate between the shapes of digital phantoms. The formula for each shape descriptor is listed to the left and each evaluated shape appears at the top of the heat map. Values were computationally determined in FIJI from regions of interest of each shape with perimeter points interpolated to a 1-pixel interval on shapes in a 512 × 512 resolution image. Resolution sensitivity analysis for each shape is included in the supplementary material. (d) Schematic of spheroid/organoid shape factor analysis workflow. The focus of the analytical methods presented in this manuscript is shape factor quantification of pre-segmented regions of interest (orange). (e) Confocal maximum projections of collagen embedded spheroids (F-actin-green, DAPI-blue) with the thresholded and segmented ROIs displayed in black and the annotated ROIs below (major axis—blue, minor axis—cyan, convex perimeter—green, and fit ellipse—magenta). (f) Heat maps present the measured values of the shape descriptors for each spheroid sample in (e).

Shape factors are dimensionless numbers that quantify object shape using parameters such as perimeter, area, convex area, convex perimeter, major axis, and minor axis^30^ [Fig. 1(a)]. Because they are size-independent, shape factors can compare structures across different length scales. For example, the widely used image-analysis software FIJI^27^ computes the following shape factors (termed shape descriptors in FIJI): aspect ratio, roundness (inverse of aspect ratio), circularity, and solidity^31^ whose respective formulas are displayed in Fig. 1 although the nomenclature and formulas can differ for other software programs. While not included in the FIJI shape descriptors, convexity is another metric that can be obtained by dividing the convex perimeter by the perimeter^32^ using a FIJI macro (https://github.com/BrittanySchutrum/FIJI-Spheroid-Morphological-Signatures). These different metrics vary in scale: roundness, circularity, solidity, and convexity range from 0 to 1, whereas aspect ratio ranges from 1 to infinity.

We first designed ten digital phantoms to evaluate how each FIJI shape descriptor classifies varied morphological features [Fig. 1(b)]. These digital phantoms were divided into four groups [Fig. 1(c)]: the first four (circle, oval, triangle, and square) have smooth contours but vary in bulk geometry; 5–7 (star, X, burst) feature surface irregularities with differing numbers and sizes of protrusions; 8–10 (half-moon, single bud, and double bud) mimic varying convexity and budding or lobular structures; and 11–14 contain varying protrusion-to-diameter ratios to mimic small (tips) or large invasions (fingers). As a reference shape, the circle scores 1 for all metrics. High convexity shapes with smooth contours (circle, oval, triangle, and square) all have perfect values of 1 for convexity and solidity because convex area equals area and the convex perimeter equals perimeter [Fig. 1(c)]. These smooth contour shapes highlight that circularity and roundness are not interchangeable metrics: circularity depends on the ratio of area to perimeter, while roundness depends on the ratio of area to major axis (i.e., elongation). Given that a circle is the smallest perimeter for a given area, the deviation from a circle, either through elongation or additional polygonal sides, causes a decrease in circularity. Mathematically, the roundness of a square is 1, identical to a circle, because in both cases the major and minor axes are equal. This counterintuitive result contrasts with the general use of round as a term to describe a shape with no sharp edges.

Next, we compared shapes that differ in the number and sizes of protrusions [shapes 5–7, Fig. 1(b)], but exhibit a roundness and aspect ratio close to 1. The convex areas of these shapes were roughly twice as large as their respective areas, resulting in similar solidity values of approximately 0.5. Hence, solidity is not a robust measure to quantify shapes with varying numbers and sizes of protrusions. Instead, circularity and convexity are more appropriate metrics to describe these differences, as both are strongly influenced by the shape's perimeter, which increases with the number of protrusions. This increase in perimeter relative to the area and convex perimeter leads to corresponding changes in circularity and convexity, respectively. Finally, we investigated alterations to a circle with a concave region, a single bud, and a double bud (shapes 8–10). As expected, roundness and aspect ratio were ineffective at quantifying these morphological features of interest, reporting only increased elongation of the shape but not other morphological changes. Because shapes with buds have increased perimeter, they are best quantified with circularity. Although concave regions are present in all these shapes, the decrease in convexity is less pronounced in shapes 8–10 than in shapes with a greater number of protrusions (shapes 5–7) [Fig. 1(c)].

Collectively, these results suggest that circularity is best suited to quantify spheroids/organoids with isolated invasion tips or points, owing to its greater sensitivity to increases in perimeter relative to increases in area. Solidity and convexity, which detect deviations from the ideal convex contours, are less suited to describing such shapes. Finally, roundness and aspect ratio can detect bulk elongation of a shape but are agnostic to corners/points/invasion tips and, therefore, not a strong descriptor of the types of invasions formed by matrix-embedded spheroids/organoids [Fig. 1(b)]. An image-resolution sensitivity analysis shows that measured shape descriptors remain consistent across different image sizes and pixel counts along the shape perimeter, validating their size independence and suitability for comparisons across length scales (supplementary material Fig. 1).

In research settings, invasive spheroids/organoids typically exhibit distinct invasion tips or fingers, which can be detected by segmenting regions of interest (ROIs) from micrographs collected by different imaging modalities, including bright-field, confocal, and optical coherence microscopy [Figs. 1(d) and 1(e)]. Our digital phantoms with varying protrusion-to-diameter ratios (shapes 11-14) demonstrate decreased circularity, solidity, and convexity with an increased protrusion to diameter ratio, although the effect was greatest for circularity [Fig. 1(c)].

Next, we evaluated how the different shape descriptors characterized invasions in experimental images. To this end, we chose three representative sample spheroids for demonstration purpose due to their marked variations in morphology and invasion: round, noninvasive; elongated, noninvasive; and round, invasive [Figs. 1(e) and 1(f)]. All spheroids were stained with phalloidin to visualize the F-actin cytoskeleton, imaged with confocal microscopy, 3D data processed into maximum projections, and threshold segmented in FIJI into ROIs for shape factor analysis [Figs. 1(e) and 1(f)]. Circularity, solidity, and convexity all decreased for the round, invasive spheroid relative to its round, noninvasive, and elongated, noninvasive counterparts [Fig. 1(f)]. Similarly, circularity, solidity, and convexity decreased for the elongated, noninvasive vs round, noninvasive spheroid, but were accompanied by a much more marked decrease in roundness relative to the round, invasive spheroid. Correspondingly, aspect ratio for the noninvasive, elongated vs both noninvasive and invasive round spheroids increased [Fig. 1(f)]. Collectively, trends from our comparison of shape factors—using both digital phantoms and experimental images—suggest that circularity and convexity are best suited to quantitatively differentiate noninvasive spheroids from invasive spheroids. In contrast, although roundness can capture sample elongation, results from quantitative analyses of phantoms and spheroids indicate that it is not a suitable metric for invasion. Since the aspect ratio is mathematically defined as the inverse of roundness, we have excluded it from our analysis from here on, as it provides no additional distinctive shape descriptor value. We note that these initial observations were conducted with a relatively small dataset, and future validation with a larger dataset is indicated to confirm relevance for future high-throughput applications.

### Extending shape factor analysis to cancer organoids

Our analyses above suggested that different shape factors vary in their capacity to capture distinct morphological features, which not only affects the analysis of invasive spheroids, but is particularly pertinent when studying organoids. More specifically, organoids often exhibit concave regions associated with local folding/buckling or protrusions caused by nonsymmetrical growth, which can be challenging to detect. To investigate the capability of each shape factor to assign quantitative morphological scores to organoids, we analyzed endometrial epithelial and oviductal tubal epithelial organoid cultures.

**FIG. 2.**
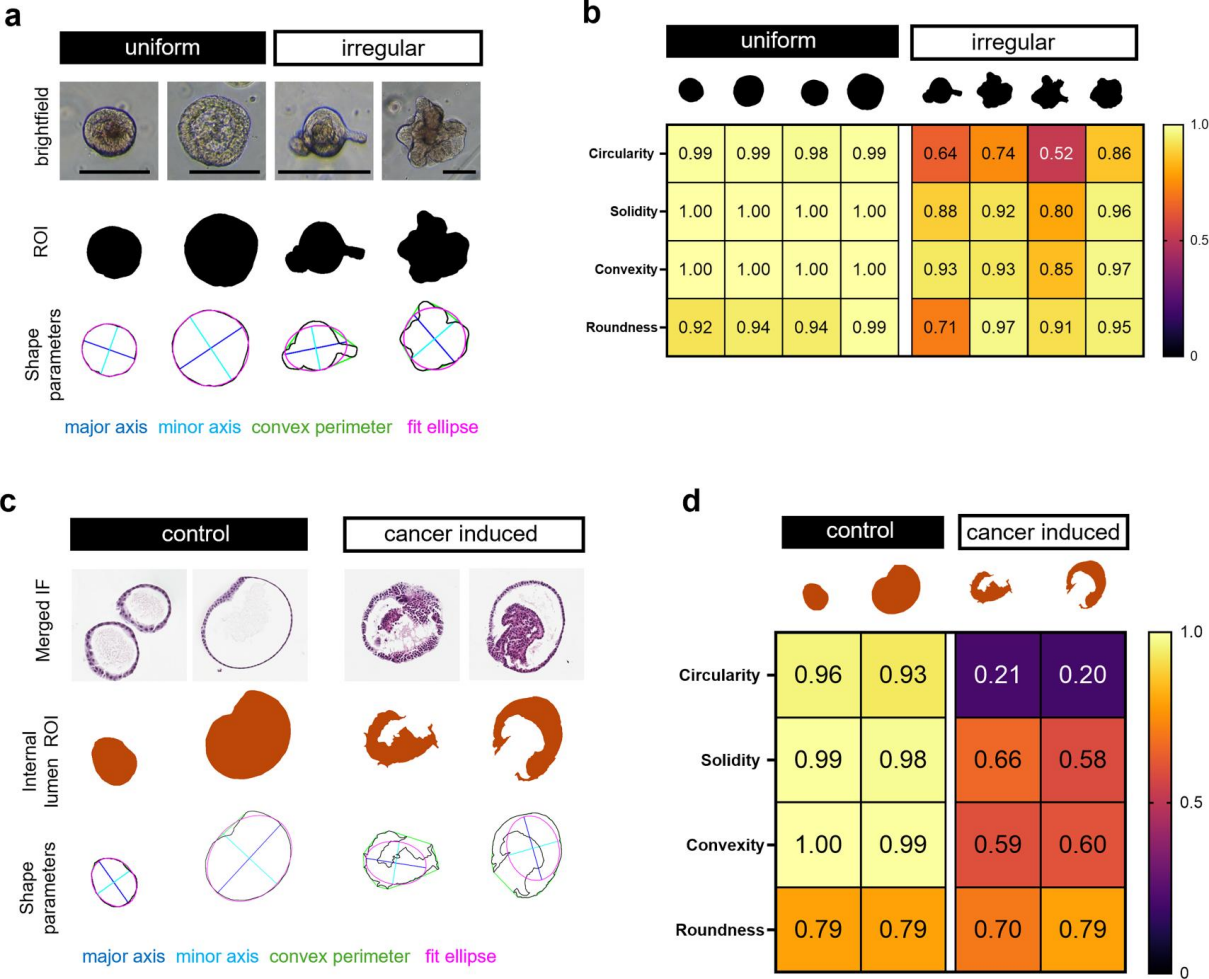
Shape factor analysis of organoids. (a) Bright-field images of non-fixed, Matrigel-embedded endometrial epithelial organoids categorized as uniform or irregular and their corresponding manually segmented ROIs, as well as shape parameters for shape factor calculations. Scale bars = 150 *μ*m. (b) Heat map showing how FIJI-based shape factors vary between uniform and irregular organoids. (c) H&E-stained cross sections of control or cancer mouse oviductal tubal epithelial organoids. Internal lumens were manually segmented and shape parameters quantified to identify differences in luminal space morphology. All sections were scanned with the ScanScope CS2 (Leica Biosystems, Vista, CA) with the doublet inserted for 40× objective magnification) cropped images are displayed here for visualization. (d) Heat map showing how FIJI-based shape factors of internal lumens vary between control and cancer-induced tubal epithelial organoids.

Endometrial cancer organoids were formed by isolating endometrial epithelial cells from the mouse uterus of *Trp53* and *Rb1*-floxed mice, treating them with adenovirus-Cre, and culturing them in Matrigel. Bright-field microscopy of non-fixed samples revealed that some organoids exhibited uniform circular shapes while others formed irregular shapes with protrusions, folds, or microlobules [Fig. 2(a)]. Manual segmentation of four representative organoids of each category yielded ROIs for subsequent analysis. Interestingly, circularity was best able to differentiate irregularly shaped from uniformly shaped organoids as it detected the largest numerical differences between both categories [Fig. 2(b)]. In contrast, solidity, convexity, and roundness were much less effective at identifying shape differences between conditions in these dataset [Fig. 2(b)].

In addition to their gross morphologies, the size and structure of organoid lumens are also important as they can, for example, be indicative of malignancy. To test how well the different shape factors can detect cancer-related changes in organoid lumen morphology, carcinogenesis was induced in oviductal tubal epithelial cells via infection with adenovirus-Cre that inactivated *Trp53* and *Rb1* prior to organoid formation while control cells were infected with a blank adenovirus. Microscopic imaging of H&E-stained organoid sections revealed clusters of cells inside the lumen of cancer organoids but not controls [Fig. 2(c)]. Inspired by prior studies quantifying lumen shape,^33,34^ we manually segmented the internal cell-free lumens to generate ROIs for shape factor analysis. Due to the accumulation of cells, lumens of cancer organoids had concave structures, which predictably yielded lower values of convexity and solidity than the control organoids with convex lumens [Fig. 2(d)]. Circularity was best able to quantify differences between the control and cancer organoid lumen shapes given the increase in concave vs convex lumen perimeter [Fig. 2(d)]. Of note, roundness did not change between the control and cancer organoids because the lengths of major and minor axes remained relatively unaffected by luminal cell growth. These quantitative results suggest that circularity may be the most universal ImageJ/FIJI shape factor to quantify changes in organoid morphology, but depending on the morphological features of interest (particularly folds and intra-luminal growths), solidity and convexity could also be of value. Integrating shape factor analysis into the analysis pipeline of organoids could help identify how organoid morphologies are determined by their genetic and lineage profiles,^35^ potentially establishing a more easily accessible metric to characterize organoids in high-throughput settings.

### Investigating the capabilities and specificity of radial length analysis

**FIG. 3.**
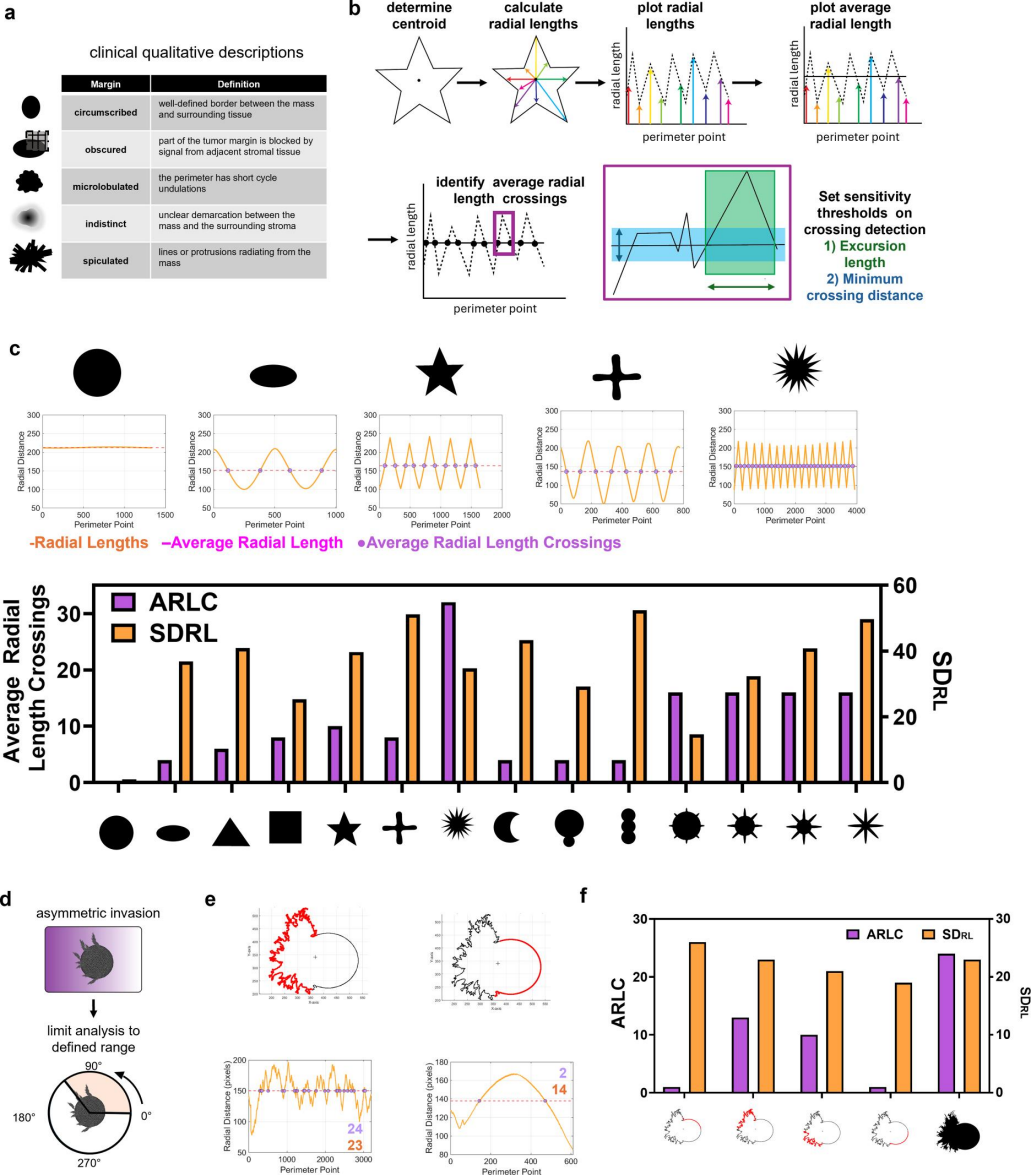
Evaluating radial length analysis as a quantitative shape descriptor on digital phantoms. (a) Summary of clinical descriptors of breast tumor margins from the BI-RADS^©^ system. (b) Workflow of the computational methods used to determine variations in radial length and the number of average radial length crossings, including a visual depiction of the sensitivity parameters that can be adjusted for each dataset. (c) Application of the radial length analysis to digital phantoms and their corresponding radial length plot. The radial length vectors are displayed in orange with the average radial length shown in magenta and the average radial length crossings shown as purple points on each plot. A bar plot displays the number of average radial length crossings (ARLC) and the standard deviation of the radial lengths (SD_RL_). (d) Schematic of segmented radial length analysis where angles can be set to analyze a defined radial segment of the perimeter. (e) Analysis of two segments of a digital phantom of asymmetric invasion; (265°–85°, left) and (85°–265°, right). The segment of the spheroid to be analyzed is highlighted in red in the generated ROIs plots (top row), and the radial length plots are displayed below (bottom row) with ARLC annotated in purple and SD_RL_ annotated in orange text. (f) ARLC (purple, left axis) and SD_RL_ (orange, right axis) for 4 quadrants of the phantom plotted from left to right: 0°–90°, 90°–180°, 180°–270°, and 270°–360°and the whole ROI 0°–360°.

Although the results above suggest that certain basic shape descriptors are well-suited to characterize changes in spheroid invasion and organoid morphology, they also detect confounding noninvasive geometric features (elongation, concave regions, single large protrusions). Therefore, we sought to identify a metric more specific to the invasive tips observed in collectively invading spheroids. Based on the irregularities of their margins, radiologists categorize breast tumors into five main classes using the BI-RADS^©^^12^ system [Fig. 3(a)]. To describe these margin irregularities quantitatively,^21^ several approaches have been developed, including analysis of radial lengths described in 1993 by Kilday *et al.*^29^ In this method, radial lengths are defined as the Euclidean distances from the center of the tumor to each boundary coordinate of the segmented shape. Variations of this radial length analysis have been used on multiple types of clinical breast or liver images.^36–40^ To evaluate the potential of radial length analysis to detect morphological changes of *in vitro* cancer models, we have developed an analysis pipeline in FIJI and MATLAB that quantifies the standard deviation of radial lengths (SD_RL_) and the number of average radial length crossings (ARLC) in spheroids.

To illustrate the fundamentals underlying radial length analysis, we generated a fictional ROI of a star [Fig. 3(b)]. In practice, ROIs are generated via automated or manual segmentation of the spheroid/organoid from images in FIJI and saved as the “.roi” file type. Subsequently, ROIs are imported into MATLAB,^41^ the centroid of the shape is computationally determined, and radial lengths are found by calculating the Euclidean distance between the centroid and each point on the shape perimeter [Fig. 3(b)]. The resulting radial length vector is plotted as a function of perimeter point index along with the computationally determined average radial length. The number of crossings or intersections between the two lines is determined and reported as the number of ARLC [Fig. 3(b)]. This value corresponds geometrically to the number of undulations at the shape margin with respect to the centroid of the shape. The criteria for determining a crossing can be set and optimized depending on the noisiness of the dataset. The two parameters that can be tuned are (1) the excursion length, which represents the number of consecutive perimeter points that must fall either above or below the average radial distance, and (2) the minimum crossing distance, which describes how far above/below the average the radial length vector points must be to count as a crossing [Fig. 3(b), supplementary material Fig. 2]. Finally, the SD_RL_ are calculated and reported as a metric indicating perimeter variations through either protrusions or an asymmetric/elongated shape. While ARLC are size independent, SD_RL_ are dependent on the resolution and/or scale of the image [supplementary material Fig. 3(b)] with default units of pixels unless converted back to length units as encoded by the original image.

To assess the capabilities of radial length analysis, we first applied the code to analyze the digital phantoms from Fig. 1 [Fig. 3(c), supplementary material Fig. 3]. This analysis provided the following insights: (1) ARLC and SD_RL_ are independent metrics which combine to provide comprehensive quantification of shape geometries; (2) ARLC increase with membrane irregularities, but also with sharp corner geometries such as the square; (3) SD_RL_ increases with deviation from a circle either in higher aspect ratio or in protrusion length/number [Fig. 3(c)]. ARLC are not sensitive to differences in protrusion lengths for the tested geometries, but SD_RL_ increases with increasing protrusion length, indicating that both metrics should be combined when quantitatively classifying objects and thus, 3D culture models, using this approach [Fig. 3(c)]. We found that for a given excursion length and minimum crossing distance, the number of ARLC was independent of image resolution while SD_RL_ increased proportionally to resolution in agreement with the size (in number of pixels) dependence of SD_RL_ [supplementary material Fig. 3(b)].

Another aspect to consider is that asymmetric radial invasion [Fig. 3(d)] may occur in the presence of biochemical or physical microenvironmental gradients, driven by processes such as chemotaxis, durotaxis, or directed migration in response to matrix microarchitecture.^42–45^ To optimize our pipeline to address this possibility, we adapted our code to analyze user-defined radial segments or “pie slices” of the ROI [Fig. 3(d)]. In this analysis, 0 degrees is set at the 3 o'clock position of the ROI, and angles increase counterclockwise. We generated nonsymmetrically invading digital phantoms [Fig. 3(e), supplementary material Fig. 4], analyzed 180-degree segments highlighted in red, and observed a higher number of ARLC in the irregular half [Fig. 3(e), left] when compared to the half with smooth surface contours [Fig. 3(e), right]. This example also highlights a notable difference in the perimeter points required to define each half with over three times as many points required for the irregular left side of the phantom [Fig. 3(e)]. Splitting the phantom into 90-degree quadrants reveals consistently high SD_RL_ across quadrants, but elevated ARLC values are limited to the quadrants containing irregular surfaces [Fig. 3(f)]. In contrast, the whole phantom ROI [360 degrees perimeter, Fig. 3(f)] has both elevated ARLC and SD_RL_ which obscures the additional directional information that is gained from a segment analysis. Therefore, regions of non-radially symmetric spheroids/organoids should be isolated to investigate spatial variance in invasion patterns to gain additional directional information.

**FIG. 4.**
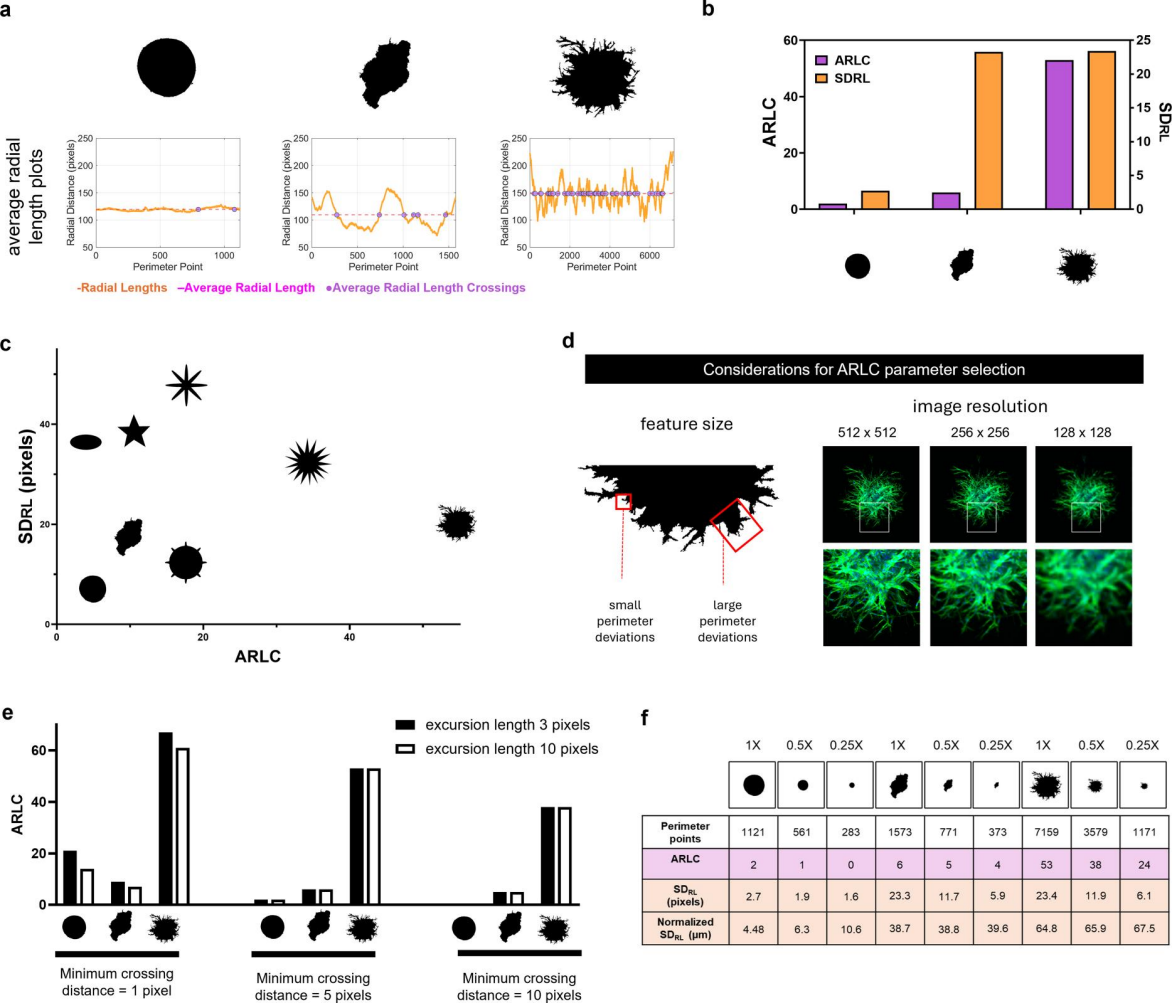
Applying radial length analysis to spheroids. (a) Radial length analysis of sample spheroid ROIs demonstrates an ability to quantify border irregularities to separate noninvasive spheroids (left, middle) from invasive spheroids (right). The radial length vectors are displayed with orange with the average radial length shown in magenta and the average radial length crossings shown as purple points on each plot. (b) Bar plot displays the number of average radial length crossings (ARLC, left axis) and the standard deviation of the radial lengths (SD_RL_, right axis,). ARLC results obtained using a minimum excursion length of three points and a minimum crossing distance of 5 pixels over the full 360°. (c) Relationship between ARLC and SD_RL_ for representative spheroids in A, B and for selected digital phantoms. (d) Considerations for parameter selection in ARLC analysis when applied to experimental datasets. (e) Effect of excursion length and minimum crossing distance on ARLC. (f) Isolated ROIs scaled down to model reduced resolution and therefore a reduction in the number of discrete points interpolated in the ROI perimeter.

### Evaluating and optimizing ARLC analysis for quantification of spheroid morphology

After proof-of-concept testing with the digital phantoms, we next performed radial length analysis of the experimental spheroids shown in Fig. 1. We found that in invasive spheroids, both ARLC and SD_RL_ increased, but in elongated, noninvasive spheroids, only SD_RL_ increased [Figs. 4(a) and 4(b)]. To further illustrate that ARLC can vary independently from SD_RL_, we plotted SD_RL_ as a function of ARLC for various digital phantoms and experimental spheroids [Fig. 4(c)]. Next, we constructed a correlation matrix to better understand the directional relationship between ARLC, SD_RL_, and the FIJI shape descriptor pairs to identify similarities/differences between metrics. This analysis was performed using a dataset of 94 brightfield images of symmetrically invading spheroids. It revealed a strong negative correlation between circularity and ARLC (r = −0.94, p < 0.0001), solidity and ARLC (r = −0.92, p < 0.0001), convexity and ARLC (r = 0.94 p < 0.0001) (supplementary material Fig. 8); ARLC was not correlated with roundness. Interestingly, ARLC correlated strongly positively with SD_RL_ (r = −0.87, p < 0.0001) in this specific dataset.

Adjusting the excursion length and minimum crossing distance parameters alters the signal to noise ratio and sets a minimum crossing threshold that must be tuned to the scale of protrusions for a specific experimental system or imaging resolution [Fig. 4(d)]. Users must critically consider which parameters are most suitable for their dataset to prevent bias in crossing detection. For example, decreasing the minimum crossing distance increased the detected ARLC [Fig. 4(e)]. Increasing both the minimum crossing distance and the excursion length lowered ARLC as the sensitivity of crossing detection is reduced; however, the trends between conditions are generally preserved [Fig. 4(e), supplementary material Fig. 5]. The resolution of the image or more specifically the number of pixels with respect to spheroid features can independently change the ARLC and SD_RL_ [Fig. 4(f)] and users are cautioned to use images of consistent resolutions when making direct comparisons. One limitation of ARLC is that the number of crossings is based on the average radial length which is impacted by asymmetry, for example in cases of unidirectional invasion, large single protrusions, or overall shape elongation (supplementary material Fig. 6). Using angle segments specifically for regions that lie entirely above/below the average radial length can reveal local membrane irregularities that are lost when analyzing the full 360 degrees of the ROI [supplementary material Fig. 6(c)]. Certain biological questions or organoid shapes may benefit from quantifying confocal cross-sections instead of maximum projections as used in Figs. 1 and 4. To demonstrate this potential application, we separately segmented ROIs from individual confocal images [10 *μ*m slices, 50 *μ*m between images shown sequentially in supplementary material Fig. 7(a)]. Results reveal small depth-dependent fluctuations in shape descriptors [supplementary material Fig. 7(b)] with more prominent changes in ARLC at discrete depths vs the maximum projection [supplementary material Fig. 7(c)]. Our pipeline can be readily used to quantify ARLC of whole spheroids or optical slices of radially invading spheroids. Here, we only present data taken in XY imaging planes [supplementary material Fig. 7(b)], however, spheroids invade in the Z direction and invasive tips pointing directly in the Z direction are not detected. Given permissive imaging conditions and successful preprocessing of XZ or YZ reconstructions to obtain ROIs in the Z direction, it will also be possible to quantify invasion in the Z direction with shape factors analysis.

**FIG. 5.**
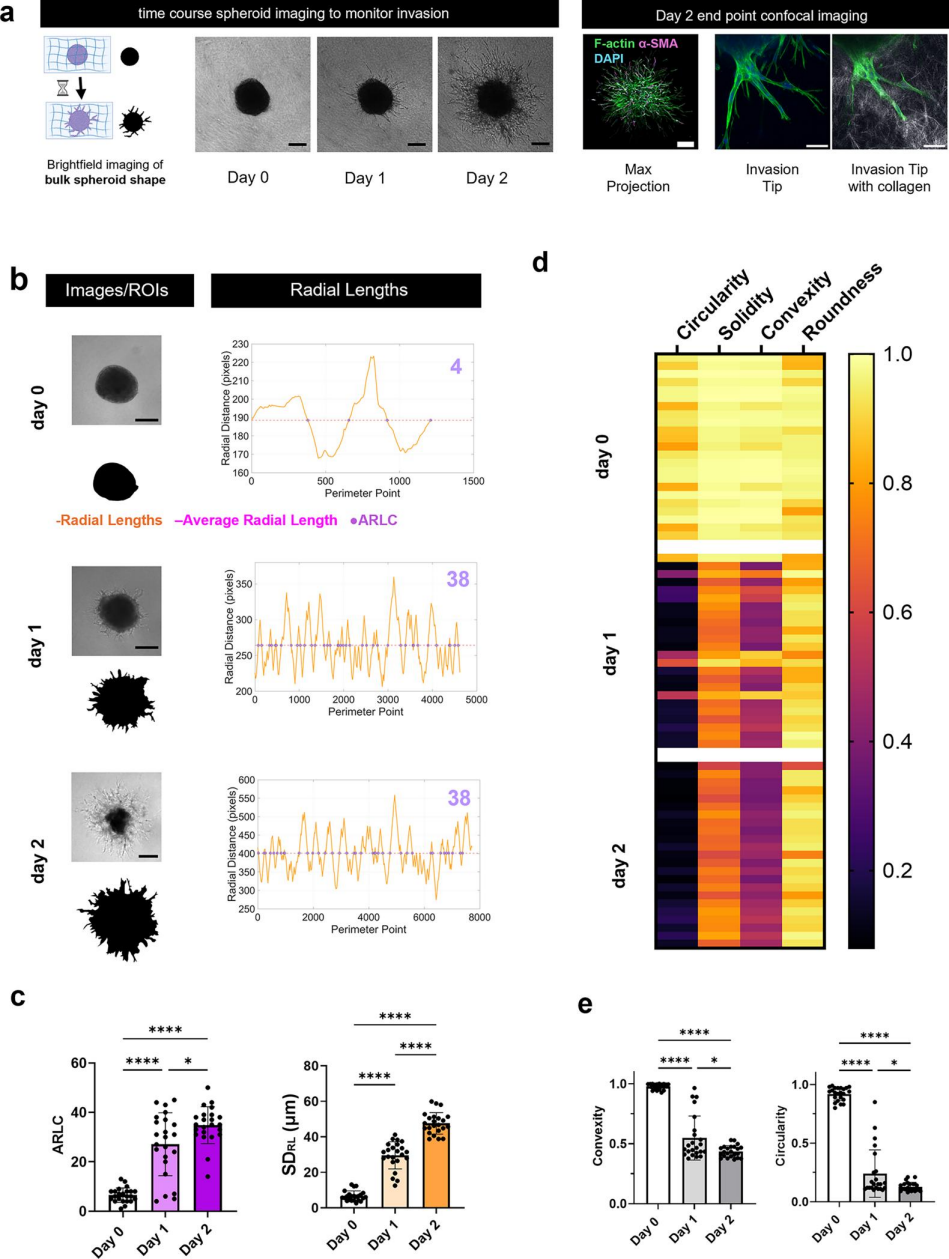
Longitudinal analysis of spheroid invasion with shape factor analysis. (a) Schematic and representative images of spheroids prepared from adipose stromal cells and embedded into collagen type I hydrogels. Spheroids were imaged with bright-field microscopy every day to monitor invasion over time (left panel scale bar = 200 *μ*m). Confocal images of fixed samples on day 2 reveal cellular-level insights into collective cell invasion tips and migration through the surrounding hydrogel at the end point of the experiment. Scale bar = 200 *μ*m max projection, =50 *μ*m in the invasion tip zoom view. (b) Spheroid ROIs were isolated from day 0, day 1, and day 2 images to quantify changes in shape factors over time during the 48 hours of culture. Representative images and their corresponding ROIs and radial length plots are displayed. Crossing sensitivity set to a minimum excursion length of 3 and a minimum crossing distance of 5 pixels. (c) Quantification of the ARLC and SD_RL_ shows a significant increase when compared to day 0 in both parameters after both 1 and 2 days of culture. Statistical significance determined by Brown–Forsythe and Welch ANOVA with Dunnett's T3 multiple comparisons test (**** = p < 0.0001, * = p < 0.05, n = 23). (d) Heat map display of shape factors reveals spheroid circularity, solidity, and convexity decrease after 1 day of invasion, while roundness remains constant. These trends are preserved on day 2. (e) Plot of the heat map values in (d) for convexity and circularity analyzed with Brown–Forsythe and Welch ANOVA with Dunnett's T3 multiple comparisons test (^****^ = p < 0.0001, ^*^ = p < 0.05, n = 23). Schematic in (a) created in Biorender.com.

**FIG. 6.**
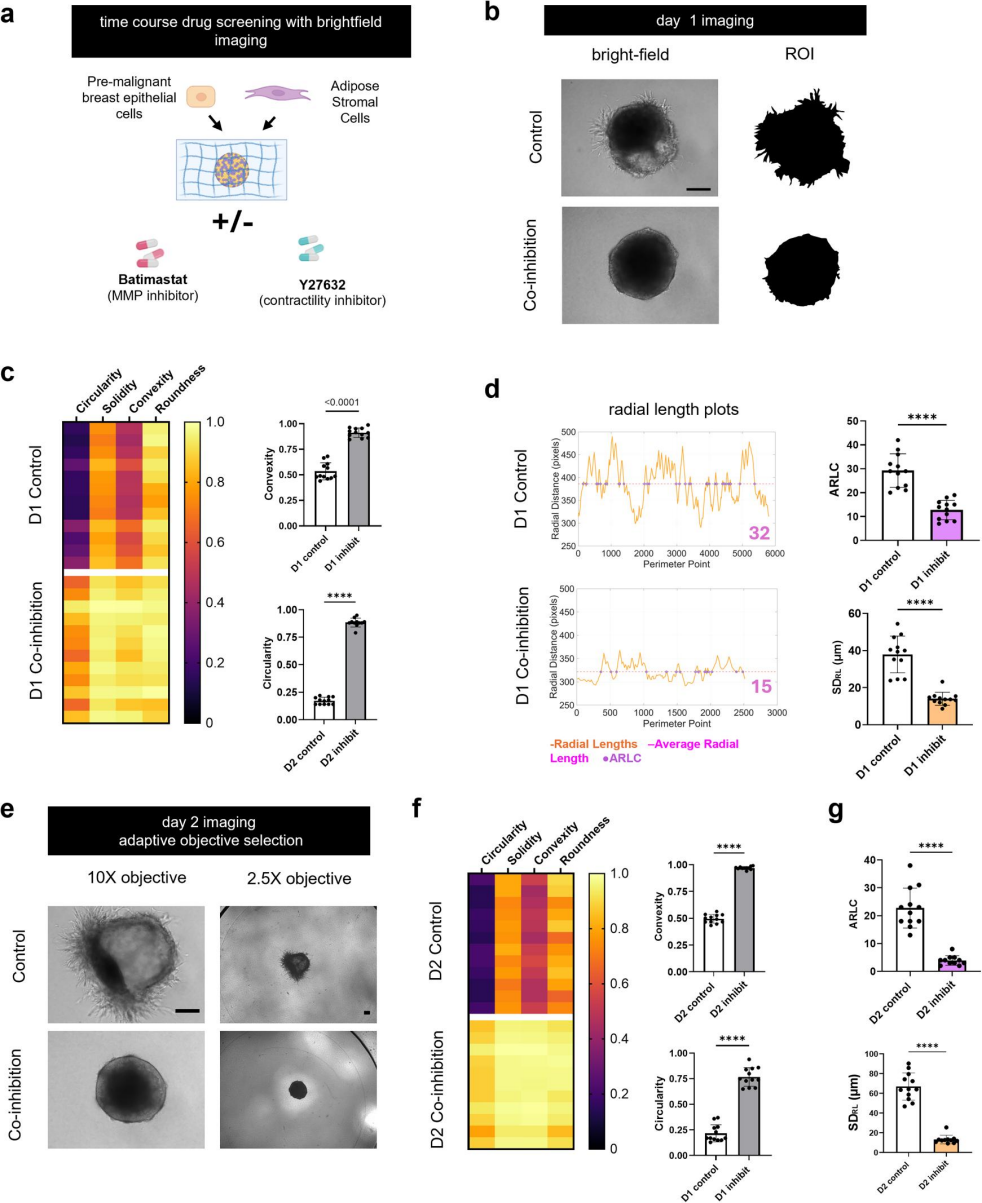
Radial length analysis of drug-treated spheroids identifies differences in early invasion independently of objective magnification. (a) Schematic of the experimental setup of collagen embedded co-culture spheroids of premalignant breast epithelial (MCF10AT1) and adipose stromal cells treated with a combination of Batimastat and Y27632 to co-inhibit matrix degradation and contractility. (b) Representative images after 24 hours of inhibition and corresponding ROIs. (c) Shape factor analysis heat map demonstrates increased circularity, solidity, convexity, and roundness when spheroids were treated with inhibitors for 1 day. Statistical significance for (c), (d), (f), (g) determined via an unpaired t-test with Welch's correction (**** = p < 0.0001, n = 12). Convexity R^2^ = 0.9170, Circularity R^2^ = 0.9913. (d) Average radial length crossings and the standard deviation of the radial lengths were significantly reduced with co-inhibition for 1 day. ARLC R^2^ = 0.7365, SD_RL_ R^2^ = 0.8189. (e) Images of day 2 spheroids taken with a 10× and 2.5× objective. Scale bars = 200 *μ*m. (f) Shape descriptor analysis of day 2 spheroids with ROIs manually isolated from images taken with a 2.5× objective to ensure the whole spheroid was in the field of view. Convexity R^2^ = 0.9907, Circularity R^2^ = 0.9157. (g) Radial length analysis of the day 2 spheroid ROIs from the 2.5× objective images. ARLC R^2^ = 0.8634, SD_RL_ R^2^ = 0.9248. Schematic in (a) created in Biorender.com.

**FIG. 7.**
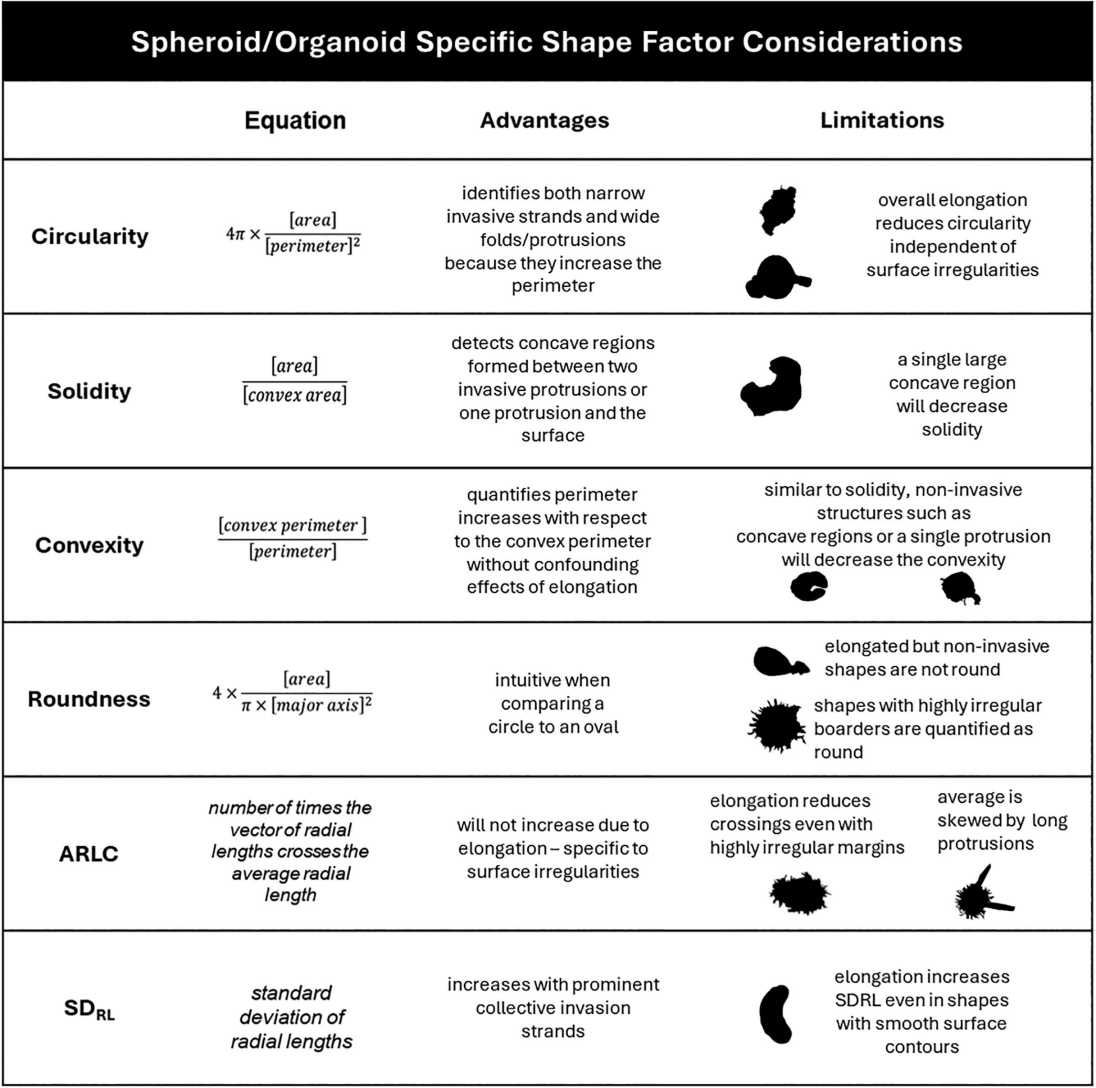
Summary table of each described shape factor detailing the mathematical definition, advantages, and limitations with visual examples of representative digital phantoms or spheroid organoid/ROIs that demonstrate the weaknesses for each metric when quantifying invasion or morphological changes.

### Live monitoring of spheroid invasion via shape factor analysis

Next, we performed shape factor analyses on a well-characterized experimental system. To interrogate the invasive phenotypes of adipose stromal cells (ASC) and their role in breast cancer progression, we previously embedded ASC spheroids into collagen hydrogels and monitored them through daily, low-resolution, bright-field imaging [Fig. 5(a), left].^19^ At the end of the experiments, we also fixed and stained the samples for high-resolution confocal or multiphoton imaging to better understand the relationship between ASC invasion and matrix remodeling [Fig. 5(a), right]. However, the latter approach is costly, inherently low-throughput, time-intensive, and prohibits longitudinal imaging of shape changes. Instead, bright-field monitoring of live spheroids at set time points during the culture period may be able to capture dynamic morphological changes and allow for longitudinal and high-throughput analysis.

To investigate if either ARLC analysis or FIJI shape descriptors could detect invasion as early as one day following spheroid embedding, we re-analyzed our previously published dataset for which we had examined invasion by simply measuring changes in spheroid area. Radial length analysis of manually generated ROIs from 23 spheroids demonstrates that ARLC and SD_RL_ of spheroids increased after 1 day in culture, enabling a more precise discrimination of emerging invasive phenotypes than our previous analysis [Figs. 5(b) and 5(c)]. On day 2, ARLC were less pronounced from day 1, but we observed an increase in the SD_RL_, which could explain the change in spheroid area between days 1 and 2 that we detected previously.^19^ Similarly, basic shape factor analysis reveals striking differences emerging as early as day 1, with decreases observed across all metrics except for roundness [Fig. 5(d)]. Circularity and convexity mirror the trends observed in ARLC with statistically significant differences between day 0 and 1 and between days 1 and 2 [Fig. 5(d)]. In summary, ARLC, circularity, and convexity all detect day 1 differences, highlighting the utility of shape factor analysis for longitudinal analysis of invasion in non-fixed samples via bright-field microscopy.

### Shape factors analysis supports time-resolved drug screening studies

Finally, we evaluated if shape factor analysis could be used to detect how a model drug regimen suppresses spheroid invasion. In our previous work, we treated co-culture spheroids of pre-malignant breast cancer cells and ASCs with Batimastat, a matrix metalloproteinase inhibitor, and Y27632, a contractility inhibitor^19^ [Fig. 6(a)]. Endpoint imaging showed that ASCs promoted invasion of pre-malignant breast cells, but treatment with the drug combination prevented invasion.^19^ These results were obtained by evaluating changes in spheroid area using images obtained by bright-field microscopy, but the detected differences were relatively small. Revaluating the dataset by creating ROI masks generated at day 1 visually illustrates the striking reduction in perimeter irregularities in inhibitor-treated spheroids (Y27632 and Batimastat: 2194 ± 158 *μ*m; Control: 4862 ± 1070 *μ*m; n = 12) [Fig. 6(b)]. All tested shape descriptors quantify this difference between control and co-inhibition; however, circularity and convexity quantitatively discriminate the conditions the most [Fig. 6(c)]. These results can be interpreted as drug treatment preventing the formation of collective invasion tips that increase the perimeter (decreasing circularity) and create concave regions (decreasing convexity and solidity). Additionally, uninhibited asymmetric invasion can lead to spheroid elongation (decreased roundness). As previously determined, these metrics are not specific to invasion and are impacted by confounding variables and morphological heterogeneity. The invasion specific radial length analysis revealed statistically significant reductions in ARLC and SD_RL_ with drug treatment at day 1 [Fig. 6(d)]. The advantage of the dimensionless nature of shape factor analysis also allows for comparisons across time points and with imaging setup variation in objectives, resolutions, and instruments [Fig. 6(e)]. This is true for all metrics used here except for SD_RL_ which does have units that correspond to physical dimensions (in this case *μ*m). Comparison across length scales is particularly advantageous when spheroids invade out of the field of view of the initial imaging objective, mandating adjustment of image acquisition parameters. For example, in the experiment shown in Fig. 6(e), control spheroids on day 2 had to be imaged at lower magnification as compared to days 0 and 1 due to their extensive invasion [Fig. 6(e)]. In this setup, the difference in optical zoom changed the number of pixels per spheroid in effect changing the resolution of that spheroid. The relative insensitivity of the FIJI shape descriptors and ARLC to changes in pixel resolution at this scale (supplementary material Figs. 1 and 3) allows for comparison of measurements between imaging sets even when they were acquired with different objectives. Analyzing the low magnification day 2 samples show the same trends as the higher magnification and resolution images on day 1. The dimensionless nature of ARLC allows day 1 values [Figs. 6(c) and 6(d)] to be directly compared to day 2 values in [Figs. 6(f) and 6(g)] despite the differences in image acquisition parameters. Overall, shape factor analysis identified drug-dependent differences in invasion earlier than simply analyzing spheroid area, opening the possibility of additional high throughput data collection and analysis in future studies.

## DISCUSSION AND CONCLUSION

Linking tissue shape and structure to biological function remains a challenge across both clinical practice and bench science. Radiologists undergo extensive training to discriminate and score subtle shape differences in clinical imaging. Researchers studying spheroids and organoids would benefit from comparable expertise but currently lack standardized frameworks for reliably interpreting morphology. As organoid and spheroid models become central to disease modeling and pre-clinical screening,^9^ scalable, quantitative methods to interpret complex morphology are required. Inspired by computer-assisted breast imaging technologies, we sought to apply dimensionless shape factor analysis to the morphological quantification of 3D *in vitro* model systems through a workflow in FIJI and MATLAB. We first developed a series of digital phantoms to serve as a visual guide to demonstrate which shape factors detect specific target morphologies. Next, we provided multiple experimental examples to assist users in selecting which metrics may be best suited for future interpretation of their own biological datasets.

The popular biology-focused image analysis software of FIJI and CellProfiler^28,46^ include a subset of user-friendly dimensionless shape factors that allow comparisons across length scales, imaging modalities, and variation in experimental conditions. While FIJI was the primary analysis tool used here, comparable analysis can be carried out in CellProfiler as demonstrated in supplementary material Fig. 9. There are a few notable differences when considering CellProfiler measure size/shape outputs: FormFactor is equivalent to Circularity in FIJI, Compactness is the inverse of FormFactor, and Eccentricity is most similar to a quantification of elongation, such as aspect ratio/roundness. To aid in comparisons between shape factors in FIJI vs CellProfiler, we have assembled a correlation matrix to identify associations between the different shape factors as computed on a sample spheroid dataset (supplementary material Fig. 9). In summary, compactness negatively correlates with convexity and circularity while positively correlating with ARLC, and Formfactor displays the opposite relationship. Regardless of the software used for computation, shape factors have successfully been used to classify single-cell morphology and serve as a predictive marker of a tumorigenic cell phenotype.^47,48^ Because of the simplicity and broad relevance, FIJI/CellProfiler shape descriptors can be readily applied beyond the subfield of cancer research. For example, in developmental biology, the shape classification of the brain,^49^ intestinal,^50,51^ or kidney^34^ organoids can provide critical information on morphogenic processes.^52^ The computational shape classifications proposed here could additionally be used in organoid experiments for disease modeling (kidney,^53^ pancreas,^54^ heart^55^), reproductive health (ovarian tissue^56^), and toxicity screening^9^ to generate hypotheses linking morphology to biological phenotype.

In the context of cancer, we have focused on multicellular spheroid invasion assays; however, shape factor analysis would be useful much earlier in the spheroid experimental workflow, e.g., to validate which fabrication method is best suited to generate uniformly shaped spheroids. Indeed, prior work has demonstrated variations in spheroid structure imparted by the fabrication method can impact gene expression and drug sensitivity.^57^ While we have focused here on applications in *in vitro* models, shape factor analysis could also prove useful in *in silico* experimental systems.^58^ Our matrix invasion analyses show that convexity and circularity can quantitatively capture shape features linked to collective invasion by identifying increased perimeter to area ratios and concave regions that would result from invasive fingers (Fig. 7). However, they are confounded by features such as a single large protrusion and may fail to detect small-scale roughness from invasion tips. Solidity, convexity, and circularity all decrease with noninvasive geometries such as elongation, an indented concave region, or a budding structure, thus limiting the interpretation of results when compared to decreased values also observed in invasion. Despite the colloquial use of round to describe a shape with no sharp corners, it is mathematically defined as the inverse of aspect ratio, capturing only elongation and making it unsuitable for most spheroid and organoid analysis applications (Fig. 7).

We created the MATLAB radial length analysis to address the FIJI shape descriptors' poor specificity for margin irregularities (Fig. 7). Our custom-built code includes tunable inputs to define the size and scale of perimeter variations that would be defined as invasive tips or fingers in a specific biological context. When applied to a dataset of collectively invading spheroids, the number of ARLC successfully differentiated spheroids based on invasiveness and roughness of the surface contours (Figs. 4–6). ARLC is best suited for identifying symmetric radial invasion or unidirectional spheroid invasion (segmented approach) in collectively invading spheroids (Fig. 7). Spheroids with predominantly single cell invasion would benefit from alternative segmentation and analysis methods to independently evaluate single cells vs the bulk spheroid core.^59,60^ Similarly, 3D cell culture systems designed for predominantly non-spheroidal unidirectional single or collective cellular invasion^61–64^ are not suited for bulk tissue-level shape factor analysis; however, applying shape factor analysis to individual cells or individual nuclei could uncover phenotypic heterogeneity among invading cells. Here, our evaluation was limited to a small subset of potential shape descriptors and a relatively small dataset of collectively invading spheroids. Given the wide variety of potential spheroid morphologies and phenotypes, the observations from our experimental system may not be generalizable to all spheroid models. Future studies using high-throughput high-content quantitative analysis of spheroid images^65^ will be needed to further validate our conclusions.

The simplicity of the ARLC analysis imposes several limitations that will require further method development and algorithm refinement. The current ARLC calculation has sensitivity limits on small perimeter undulations with respect to the overall diameter and limited interpretation of complex features such as bifurcated invasion tips. Another confounding factor is ROI elongation, whereby ARLC will decrease, and SD_RL_ will increase in an elongated shape independent of the presence/absence of surface irregularities (Fig. 7). To overcome this limitation, angular segments can be isolated and analyzed independently (supplementary material Fig. 6); however, the current MATLAB code requires user-defined segments. This manual input significantly reduces the throughput and usefulness of this approach. An alternative method to overcome the dependence on centroid location and subsequent sensitivity to elongation is to not calculate the radial length as the distance from the perimeter point to the centroid, but instead, calculate the radial deviation from a low-order contour approximation, using elliptical Fourier decomposition, which follows the bulk shape of the spheroid to emphasize protrusions across the full spheroid surface.^66^ Similarly, fit ellipse metrics such as lobulation count^67^ have been used in hepatic imaging studies and should be tested in spheroids/organoids. While adapting metrics based on elliptical Fourier decomposition or similar fit ellipse approaches is outside the scope of this manuscript, this is an active area of ongoing research for analysis improvement.

The analysis methods presented here assume pre-segmented ROIs generated from spheroid/organoid images. Because manual segmentation can introduce inter-user variability and limit analysis throughput, analysis pipelines can be greatly improved by automated organoid/spheroid segmentation assisted by deep learning or convolutional networks.^68^ Examples of preexisting segmentation and analysis tools include ilastik,^69^ INSIDIA,^65^ SpheroScan,^70^ OrganoSeq,^26^ and SpheroidJ.^71^ Brightfield images of spheroids in collagen hydrogels as featured in Figs. 5 and 6 are particularly difficult to segment with the aforementioned segment analysis tools, due to heterogeneous grayscale values within spheroids, variations in brightness within the gel backgrounds, artifacts created by capturing edges of the culture well, and limited resolution when identifying spheroid boundaries. Therefore, we opted to use manual segmentation for ROI generation here. However, as mentioned above, manual segmentation significantly reduces the throughput of the system and represents a high priority area for future improvement. Applications with high throughput systems will also require quality control/outlier removal with tools such as SpheroidAnalyseR.^72^ Future efforts to combine radial length and shape factor analysis with tools to analyze cellular and subcellular changes^19,22,73–76^ hold the potential to advance overall understanding of the biophysical and biochemical mechanisms involved in invasion. Moreover, such studies could identify morphological biomarkers that enable imaging of invasion at low resolution and magnification at multiple time points in a large number of samples as, for example, necessary in platforms with robotic liquid handling and spheroid culture^77^ for precision medicine applications.

In summary, we have analyzed the capabilities and limitations of FIJI shape descriptors and compared them with radial length analysis to detect specific morphological features of shapes. Through the analysis of digital phantoms, organoids, and invasive spheroids, we have demonstrated the potential biological applications of morphology analysis and outlined key criteria for selecting shape factors. Developing automated, interpretable shape analysis pipelines would bridge the expertise gap between clinical image interpretation and laboratory practice, improving reproducibility, accelerating translational discovery, and allowing morphology-based phenotyping at the throughput required for large-scale applications such as drug testing.

## METHODS

### Creation of digital phantoms

Phantoms were hand drawn as regions of interest (ROIs) in FIJI using the oval, polygon, or freehand selection tools on a new 512 × 512 8-bit image.^27^ ROIs were interpolated with 1 pixel interval to set the number of perimeter points. For resolution analysis, generated ROIs were scaled by 0.5 or 2 to proportionally fit 256 × 256 and 1024 × 1024 8-bit images, respectively, and interpolated at their new size. Asymmetric phantoms featured in supplementary material Fig. 6 were generated through a combination of hand-drawn original shapes and modification of experimental samples. Shapes were imported into FIJI, ROIs generated via the analyze particle tool, and interpolated with a 1-pixel interval.

### Primary mouse endometrial and oviductal tubal epithelial organoid culture

Primary cells were harvested from the mouse uterus or oviduct of *Trp53* and *Rb1* floxed mice.^78,79^ Endometrial organoids were generated from single endometrial epithelial cells cultured in Matrigel and treated with adenovirus-Cre at the time of isolation to induce a cancer phenotype. Non-fixed organoids were imaged on day 7 in the Matrigel culture. A fraction of the organoids was treated with 4 days of DBZ; however, treatment vs control conditions are not differentiated in the analysis and application in this shape factor study. Tubal epithelial organoids were generated via culture in Matrigel. For these organoids, control cultures were treated with an adenovirus blank, while cancer-induced organoids were infected with adenovirus-Cre that inactivated *Trp53* and *Rb1* in single cells on day 0 of culture. After 14 days of culture, organoids were isolated from Matrigel and resuspended in 4% paraformaldehyde (PFA) for 1 h. Fixed organoids were washed with 1× phosphate-buffered saline (PBS) three times prior to resuspending in HistoGel (Epredia, HG4000012) at 65 °C. HistoGel suspensions of organoids were set after 10 min, where they were then subjected to standard tissue processing and paraffin embedding.^79^ 4 *μ*m paraffin sections of organoids were H&E stained for imaging.

### Collagen-embedded adipose stromal spheroids, and adipose stromal-breast cancer co-culture spheroids

Murine adipose stromal cells were isolated from the mammary fat pads of wild-type mice as previously described.^19^ Human stromal cells were Adipose Derived Stem Cells (Lonza), and human breast cancer cells were the MCF10AT1 cell line or HAS3 overexpressing MCF10A cells.^62^ Cells were expanded in their corresponding media^19,62^ and formed into spheroids of 5000–10 000 cells per spheroid via aggregation on agarose-coated plates on an orbital shaker for 24 h or via the hanging drop method using media supplemented with 25cp methylcellulose and type 1 collagen and cultured for 24 h. Spheroids were embedded into 6 mg/ml collagen type 1 hydrogels and cultured for 2–3 days with bright-field imaging every 24 h. Spheroids were fixed in 4% PFA and stained with Alexa Fluor 488-labeled phalloidin and 4′,6-diamidino-2-phenylindole (DAPI) before being imaged on a Zeiss 710 or 880 confocal microscope.

### Automated generation of spheroid regions of interest from fluorescence images

Confocal maximum projections of F-actin-stained spheroids were thresholded using the triangle thresholding algorithm in FIJI to create a binary image. The analyze particles tool was used to detect and create spheroid ROIs from these binary images, which were subsequently saved in the ROI manager. Spheroid images were acquired in 512 × 512 pixel images, and ROIs interpolated to a 1-pixel interval. Detached single cells or clusters of cells were excluded from the ROIs to focus on collective invasion from the spheroid.

### Manual generation of spheroid/organoid regions of interest from bright-field images

Both bright-field images of spheroids/organoids and H&E-stained cross sections of organoids were manually traced in FIJI to generated ROIs from the source images via the polygon selection tool. ROIs were interpolated with a 1-pixel interval.

### FIJI shape descriptors

FIJI shape descriptors were added to the acquired measurement parameters by checking the shape descriptors box in the set measurements menu of the software. ROI measurements yielded results for aspect ratio, roundness, circularity, and solidity—all of which are dimensionless units. Details of shape descriptor calculations can be found in the FIJI user guide.^31^

### Convexity quantification via a custom-built FIJI macro

Convexity is not a parameter conventionally included in the shape descriptor measurements of FIJI and therefore had to be generated using a FIJI macro. Briefly, the Convex Hull function was run to generate the ROI of the convex hull of the ROI. The convex hull is added to the ROI manager, measured to obtain its perimeter, and divided by the perimeter of the original ROI. Full code is available in the supplementary material.

### MATLAB script for radial length analysis

To determine the standard deviation of radial lengths (SD_RL_) and the number of average radial length crossings (ARLC) from ROIs generated from phantoms or experimental images, a MATLAB method was developed. Briefly, FIJI-generated ROIs were first imported into MATLAB using an adapted version of the ReadImageJROI plugin^41^ originally built by Dylan Muir (https://github.com/BrittanySchutrum/ReadImageJROI/blob/master/ReadImageJROI.m). Next, the centroid coordinates of the shape were obtained from the ROI by isolating the data from the adapted ROIs2Regions function^41^ (https://github.com/BrittanySchutrum/ReadImageJROI/blob/master/ROIs2Regions.m). The centroid and all perimeter points of the ROIs were plotted on a XY plot for visualization and conformation purpose. To determine the radial length from each perimeter point (i) to the centroid, the mean squared distance was determined as follows:

$${\text{radial length}}_{i}=\sqrt{{(x_{i}-x_{\text{centroid}})\;}^{2}+{\left(y_{i}-y_{\text{centroid}}\right)}^{2}},$$

where x_i_ is the x coordinate of perimeter point i, y_i_ is the y coordinate of perimeter point i, and x_centroid_ and y_centroid_ are the x and y coordinates of the centroid, respectively. All the radial lengths were collected into a vector of the length of the number of perimeter points. The SD_RL_ was found from the standard deviation of this radial length vector. The average radial length—computed by calculating the average of the original radial length vector—was plotted as a horizontal line. The number of ARLC is computed by looking at pairs of data points—radial length_i_ and radial length_i+1_. A crossing is an instance where the radial length of the i point and i + 1 point span the average, so in the case that (1) [radial length_i_ > average radial length and radial length_i+1_ < average radial length] or (2) [radial length_i_ < average radial length and radial length_i+1_ > average radial length]. Detection of crossings can be restricted by (1) a minimum number of points on each side of the average (defined as the excursion length) and (2) a minimum distance that the radial length must cross the average radial length by (defined as the minimum crossing distance). The developed code allows restricting crossing detection by excursion length and minimum crossing distance to reduce noise and fit the pixel length scale of the individual dataset. Full MATLAB code is available in the supplementary material. Additional information and instructions are provided in the README file in the GitHub repository https://github.com/BrittanySchutrum/AverageRadialLengthCrossings-Spheroids.

### Data presentation

Heat maps and bar charts were generated in GraphPad Prism. All line plots were generated in MATLAB. Schematics were drawn using Biorender, Adobe Illustrator, and PowerPoint.

### CellProfiler analysis

For segmentation consistency across software using the same dataset, pre-segmented binary images of spheroids were imported into CellProfiler. The following sequential pipeline was used to measure CellProfiler-defined shape factors: IdentifyPrimaryObjects > MeasureObjectSizeShape > ExportToSpreadsheet. Here, we specifically investigated FormFactor, Compactness, and Eccentricity.

### Statistical analysis

All statistical analysis was done in GraphPad Prism, with the details of each statistical test provided in the corresponding figure captions.

## SUPPLEMENTARY MATERIAL

See the supplementary materials include supplemental Figures 1–9 and supplemental code 1–2.

## Data Availability

The data that support the findings of this study are available from the corresponding author upon reasonable request. The FIJI macros are available on Zenodo, Ref. 80 and the MATLAB script is available on Zenodo, Ref. 81.
